# Comparison of the Short Time Effect of an Oral Hygiene Education in Four Sessions via Quantitative Light-Induced Fluorescence Technology Versus Disclosing Agents in Children: A Randomized, Crossover Clinical Trial

**DOI:** 10.3390/children11111371

**Published:** 2024-11-12

**Authors:** Sangkyu Han, Seong Jin Kim, Taeyang Lee, Hoi-In Jung, Ko Eun Lee, Je Seon Song

**Affiliations:** 1 Department of Pediatric Dentistry, College of Dentistry, Yonsei University, Seoul 03722, Republic of Korea; sbs0272@yuhs.ac (S.H.); kseongjin@yuhs.ac (S.J.K.); 2sun@yuhs.ac (T.L.); leekoeun@yuhs.ac (K.E.L.); 2 Department of Preventive Dentistry & Public Oral Health, College of Dentistry, Yonsei University, Seoul 03722, Republic of Korea; junghoiin@yuhs.ac; 3 Innovation Research and Support Center for Dental Science, Yonsei University Dental Hospital, Seoul 03722, Republic of Korea; 4 Oral Science Research Center, College of Dentistry, Yonsei University, Seoul 03722, Republic of Korea

**Keywords:** Qscan device, disclosed plaque visualization, quantitative light-induced fluorescence, oral hygiene education

## Abstract

Objectives: The aim of this study is to compare the effectiveness of Qscan plus™ (AIOBIO, Seoul, Korea) based on quantitative light-induced fluorescence (QLF) technology and disclosing agents in oral health programs in children. Methods: A randomized crossover study was conducted for Korean children aged 6–11 years. Fifty-eight participants (29 to use Qscan plus™ first and 29 to use the disclosing agent first) were enrolled in this study. The participants were randomly divided into two groups. One group was assigned to brush with Qscan plus™, while the other group brushed with disclosed plaque visualization. One month later, the groups switched procedures. A total of 39 participants were analyzed, excluding those lost during the trial. There was no adverse event during the trial. The patient hygiene performance (PHP) index was used to assess oral hygiene status, and questionnaires about oral health behavior and attitude were completed. The data were analyzed using repeated-measure analysis of variance, with a significance level of *p* < 0.05. Results: The PHP score decreased significantly on post-brushing and follow-up compared to baseline in both methods (*p* < 0.001), but there was no significant difference between the two methods. After oral hygiene education, participants’ brushing time increased, and their oral care attitudes improved. More participants preferred the Qscan device to the disclosed plaque visualization because it is more easily noticeable. Conclusions: The Qscan device has a similar educational effect as disclosing agents, and can be used as a supplementary tool to encourage children in oral hygiene education.

## 1. Introduction

Ensuring proper oral hygiene is paramount for the prevention and control of prevalent oral diseases, such as dental caries and periodontal disease [1]. Among the various methods available for maintaining proper oral health, mechanical plaque removal, particularly through regular toothbrushing, is considered the most effective [2]. Consistent toothbrushing disrupts plaque accumulation on tooth surfaces, thereby inhibiting bacterial growth and preserving overall oral health [3].

The significance of maintaining good oral hygiene becomes particularly pronounced around the age of six when the first permanent molar begins to emerge on the gingiva [4]. This period, the transition from primary dentition to mixed dentition, is critical for developing lifelong oral hygiene habits. The research shows that first permanent molars are especially vulnerable to decay during this stage, particularly among children in rural areas, emphasizing the need for consistent and effective oral care routines. Another study shows that family-based guidance on brushing can positively influence long-term oral health outcomes by reinforcing healthy routines [5]. Under the guidance of their parents, children begin to brush their teeth independently, establishing essential daily routines and recognizing the importance of proper oral care. The habits formed during this crucial period are likely to persist throughout life, making a substantial impact on long-term oral health [6,7]. Thus, it is vital to implement thorough oral hygiene education and promote regular practice during early childhood.

Despite such education, many studies indicate that toothbrushing skills in children under the age of ten remain inadequate [8,9]. Children often lack the manual dexterity necessary for effective brushing and may not fully comprehend the anatomical structure of their teeth and gingiva. Additionally, insufficient motivation or difficulty in understanding detailed brushing instructions can further hinder proper plaque removal [10,11]. As a result, children frequently fail to adequately clean their teeth, leaving plaque behind on the surfaces.

Furthermore, dental plaque presents a significant challenge due to its subtle coloration that closely resembles the natural shade of teeth, making it difficult to detect visually [12]. Plaque often appears translucent or pale yellow, frequently going unnoticed by children during the brushing process [13]. Consequently, despite their efforts, the removal of plaque may be insufficient, potentially leading to adverse long-term effects on oral health [14]. This highlights the necessity for supplementary tools or techniques that can assist children in more effectively identifying and eliminating plaque during their daily oral hygiene practices.

In order to distinguish dental plaques from teeth easily, plaque-disclosing agents have long been used [15]. Plaque-disclosing agents disclose the biofilm and dental plaques in the oral cavity, making it easier to detect dental plaques for professional evaluation and to motivate patients [16,17]. However, there are several disadvantages of disclosing agents in dental clinics or at home: objectionable taste, risk of staining clothes or skin, and a long chair time to remove stained plaque thoroughly. Due to these disadvantages, there are some limitations to using plaque-disclosing agents in daily routine [18].

Recently, various studies about quantitative light-induced fluorescence (QLF) technology have been presented in the field of dentistry. QLF enables the visualization of dental plaque by detecting red fluorescence produced from porphyrins, which are byproducts of bacterial metabolism in the biofilm [19,20,21]. Using this technology, a portable device called Qscan (AIOBIO, Seoul, Korea) was created to facilitate easy dental plaque detection by patients. The Qscan device serves as a valuable tool in oral hygiene education, offering visual guidance and encouragement for children. Recent studies have indicated that QLF technology can significantly improve oral hygiene practices and perceptions among children and adolescents [22,23]. However, there are no studies comparing the effectiveness of using the Qscan device and disclosing agents in oral hygiene education programs so far. This paper aims to compare the effectiveness of the Qscan device and disclosing agents in oral hygiene programs in children.

## 2. Materials and Methods

A randomized, two-period crossover study was carried out from August 2019 to October 2020 in Yonsei University Dental Hospital. Informed consent was obtained from the parents or caregivers of all participants prior to the trial. The study was conducted according to the procedures and protocols approved by the Institutional Review Board at Yonsei University Dental Hospital (2-2019-0022).

### 2.1. Participants

The sample size for this study was calculated using the Gpower program (G*Power Version 3.1.9.7 statistical software). The sample size was estimated to be 42 participants at a power of 0.80, an alpha error level of 0.05, and an effect size of 0.25. Considering potential dropouts, a total of 58 children were included in this study, with 29 participants assigned to each group. At first, Seventy-two children aged 6 to 11 years with normal physical and cognitive development were evaluated for eligibility, and 58 participants met the inclusion criteria.

The inclusion criteria were:

(i) Children aged 6 to 11 years with overall good general health;
(ii) Children with fully erupted permanent first molars and central incisors.

**Figure 1:**
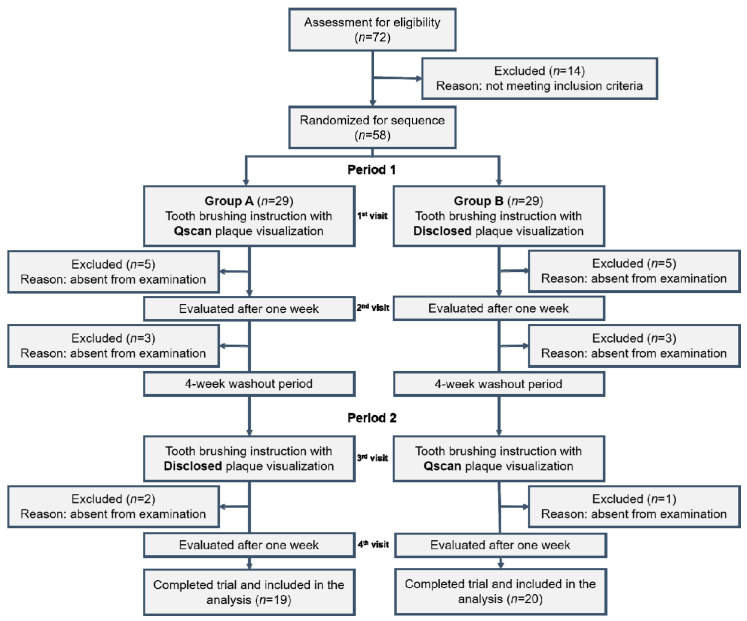
Flow diagram for enrollment in this study.

The exclusion criteria were:

(i) Children who had received oral hygiene instruction or used disclosing agents during the 3 months prior to the study;
(ii) Permanent first molars restored with stainless steel crowns or gold crowns;
(iii) Children who can’t brush their teeth themselves due to the medical conditions;
(iv) Patients who are undergoing orthodontic treatment (fixed appliance),

None of the selected 58 children had any disabilities such as mental disorders, encephalopathy, autism, intellectual disabilities, developmental disorders, or visual impairments, nor did they have cancer or severe immune diseases. Fifteen children had a mild medical history, such as rhinitis or atopic dermatitis. A total of 58 participants were randomly divided into two groups, group A and group B. Group A was assigned to brush with Qscan plus™ (AIOBIO, Seoul, Republic of Korea), which is a portable type quantitative light-induced fluorescence (QLF) device, while group B brushed with disclosed plaque visualization. One month later, the groups switched procedures. During the evaluation periods, some of the participants were absent for unknown reasons. The final numbers of subjects were 19 in group A and 20 in group B (Figure 1).

### 2.2. Plaque Assessment

**Figure 2:**
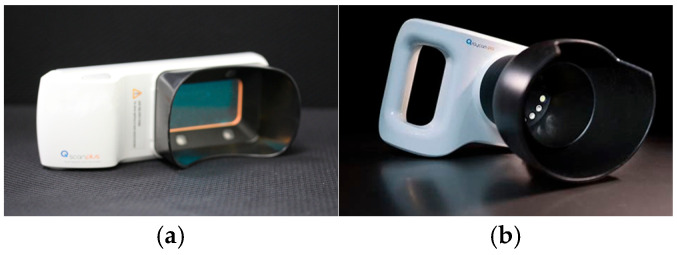
A quantitative light-induced fluorescence (QLF) device used in this study. (**a**) Qscan plus™ (AIOBIO, Seoul, Republic of Korea), (**b**) Qraycam™ Pro (AIOBIO, Seoul, Republic of Korea).

Oral hygiene was assessed based on the extent of plaque accumulation using the patient hygiene performance (PHP) index. The PHP index divides a single tooth surface into five areas: mesial, distal, and middle thirds, with the middle third further subdivided horizontally into incisal, middle, and gingival thirds. Each area was scored as 1 if plaque was present and 0 if it was absent. A single tooth could receive a score ranging from 0 to 5, and the total score for the six teeth (buccal surfaces of both maxillary first molars, maxillary right central incisor, mandibular left central incisor, and lingual surfaces of both mandibular first molars) was divided by the number of teeth to calculate the mean score [24]. The PHP score was acquired under a quantitative light-induced fluorescence (QLF) device instead of a disclosing agent because the application of a disclosing agent could affect the results when participants used Qscan plus™ (Figure 2a).

QLF-D images of the frontal view were acquired using Qraycam™ Pro (AIOBIO, Seoul, Korea) (Figure 2b), which is a camera-type quantitative light-induced fluorescence (QLF) device to analyze the simple hygiene score (SHS) and ΔR30 and ΔR120 values using a QLF-D analysis program (Q-Ray™ v 1.42; Inspektor Research Systems BV, Amsterdam, The Netherlands).

### 2.3. Random Sequence Generation and Allocation Concealment

All of the participants were randomly divided into two groups using the random function of Excel 2016 (Microsoft, Redmond, WA, USA) without blocking. Each participant was blinded to the allocation sequence until intervention was assigned.

### 2.4. Clinical Procedures

On the first day of the visit, a baseline questionnaire was administered to all participants to evaluate their oral health knowledge, attitudes, and behaviors. A single dentist gave all participants the same questionnaire. Adapted from the questionnaire created by Angelopoulou et al. [25], the survey included questions regarding the appropriate toothbrushing techniques, the frequency of brushing per day, the duration time of brushing, and the level of concern regarding their own oral hygiene. For 11-year-olds, no dentist intervention was necessary, as they could fully understand the questionnaire. For 6-year-olds, the dentist provided explanations only when they had difficulty understanding the questionnaire to help them complete it accurately. All participants were provided with fluoride-containing toothpaste and medium hardness manual toothbrushes for school-age children (AIOBIO, Seoul, Republic of Korea) throughout the whole experimental period.

An individual lesson on the appropriate frequency and method of toothbrushing was provided to each of the participants with a toothbrushing model within 10 min. The recommended brushing method for school-age children involved using the rolling technique for the buccal and lingual surfaces, a horizontal scrub method for the occlusal surfaces, and a sweeping motion from inside to outside for the lingual surfaces of the anterior teeth [26]. Additionally, the entire dentition was divided into six sextants, with a minimum of thirty seconds of brushing instructed per sextant. It was explained that participants were encouraged to brush their teeth three times a day, while instructions regarding the use of dental floss were not included separately [27]. Afterward, group A participants were taught how to use the Qscan device to check their own plaque, and independently brushed their teeth for 3 min using the device. For group B, disclosing agents (2-Tone Disclosing Solution, Earth City, MO, USA) were applied to all the participants’ tooth surfaces, and the participants rinsed out the excess solution. The educator helped the participants see the disclosed plaque in their mouths, and they brushed their teeth for 3 min. After brushing, the post-brushing PHP score was evaluated, and QLF-D images of the frontal view were acquired. Prophylaxis with a rubber cup was then performed to reduce the child’s PHP score to zero.

One week later, the participants revisited for follow-up. They were instructed not to brush their teeth 4 h before the appointment. The PHP score was evaluated, and QLF-D images of the frontal view were acquired. The participants also completed a satisfaction questionnaire about the method they had used to check on their plaques on the last visit.

Four weeks later, the participants went on the same procedure with the method switched. Group A brushed their teeth using disclosed plaque visualization, and group B used the Qscan device. The PHP score was evaluated, and QLF-D images of the frontal view were acquired at pre-brushing and post-brushing states. One week later, which was the last visit, the participants’ follow-up PHP score was evaluated, and QLF-D images were taken. They also completed the same questionnaire survey as the first visit, as well as the satisfaction questionnaire about the overall procedures.

A trained dentist and dental hygienist conducted the procedures. The dentist, unaware of the method assigned to each participant, conducted the plaque evaluations while the dental hygienist delivered the oral hygiene lessons. To ensure blinding, the plaque assessment and toothbrushing sessions were conducted in separate rooms.

### 2.5. Statistical Analysis

Statistical analyses were performed by SPSS software version 26 (IBM Corporation, Armonk, NY, USA), with a significance level of *p* < 0.05. The repeated-measures analysis of variance (ANOVA), followed by the Bonferroni post hoc test, were used in data analysis.

## 3. Results

**Figure 3:**
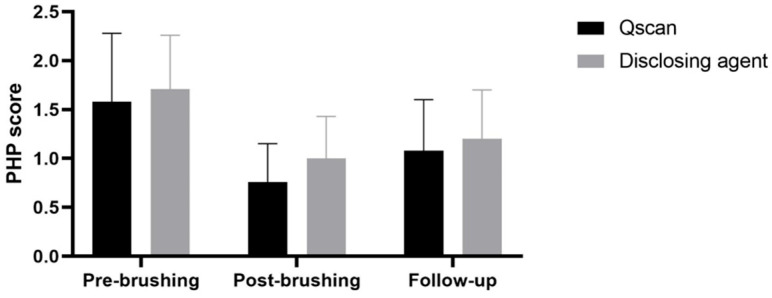
Mean and standard error of PHP score between the two methods.

The PHP score decreased significantly on post-brushing and follow-up compared to baseline in both methods (*p* < 0.001), although there was no significant difference between the two methods (Figure 3). The other variables of oral hygiene status, SHS, ΔR30, and ΔR120, decreased significantly after brushing and had no significant difference between the two methods (Table 1). The repeated-measures ANOVA results showed a significant difference over time points in PHP score, SHS, ΔR30, and ΔR120 (*p* < 0.001). However, there was no significant interaction effect between time and group in PHP, SHS, ΔR30, and ΔR120 (Table 2).

**Figure 4:**
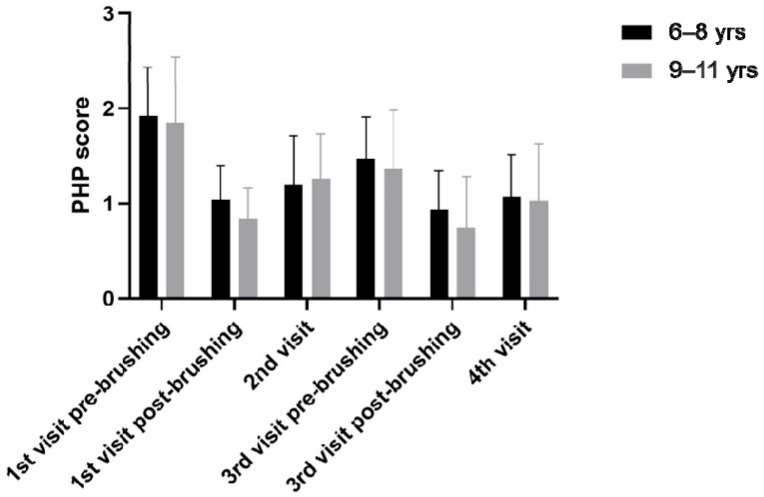
Mean and standard error of PHP score between age groups.

When all the participants were re-divided into a group of 6 to 8 years and a group of 9 to 11 years, the PHP score of both age groups decreased significantly after brushing, with no significant difference between the age groups (Figure 4).

Responses from the questionnaire survey during the first and last visits are shown in Table 3. There was no significant difference in the frequency of brushing teeth per day, which more than 80% of participants answered 2 or 3 times. Among the responses about the brushing time, there was a minor decrease of “I don’t know” from 28.2% to 17.9%; meanwhile, there was a significant increase of “3 min” from 23.1% to 41%. Among the responses about the degree of concern about oral hygiene, the responses of “none” and “low” decreased while the responses of “high” increased.

The most preferred method for toothbrushing instruction was “Qscan” (43.6%), followed by ”both” (35.9%) (Table 4). The reason for the preference for Qscan was “easily noticeable” (Table 5).

## 4. Discussion

Previous studies have displayed that disclosing agents can be used as useful adjuncts to toothbrushing instruction [28,29,30]. However, due to disadvantages such as difficulty in use, potential staining of skin or clothing, and the prolonged time required to remove all stained plaque, there is a need for new adjuncts in toothbrushing education [18]. Recently, ultrasonic toothbrushes, microscale mist units, and smart toothbrushes with mobile applications have been developed, with research demonstrating their efficacy [31,32,33]. These technologies emphasize the efficiency of plaque removal and highlight the importance of visualizing plaque, necessitating the validation of alternatives to disclosing agents. Therefore, this study aims to demonstrate the educational effectiveness of using QLF technology for visualizing plaque, which addresses the limitations of disclosing agents.

The results of this study indicate that Qscan is as effective as disclosing agents in toothbrushing instruction. Following the toothbrushing education with Qscan, the PHP score, SHS, ΔR30, and ΔR120, all decreased at the follow-up visit. While the average values of these indices were lower when using Qscan compared to the disclosing agent, the differences were not statistically significant (Table 1). Both methods led to improvements in oral hygiene indices over time, but no differences were observed between the two methods (Table 2). Thus, using Qscan for toothbrushing instruction improved oral hygiene, with no notable difference compared to the disclosing agent.

Studies evaluating children aged 6 to 12 have reported that plaque removal efficiency improves as age increases [34]. Nevertheless, a study on 12-year-olds indicated that, despite considerable efforts to help children acquire recommended brushing techniques, they still do not effectively master these methods [35,36]. There is a need to understand the factors that hinder children from following brushing recommendations. In this study, we aimed to evaluate the outcomes, focusing on simplifying plaque visualization, considering that plaque is typically not readily visible. Although the PHP score of the 6–8 years old group was consistently higher than that of the 9–11 years old group, both age groups showed similar trends in this study and showed no significant difference between groups. This result indicates that even 6–8 year olds, who are in the lower grades of elementary school, can fully improve their oral hygiene status by providing appropriate oral hygiene education.

According to the questionnaire, most participants responded that they brush teeth ‘twice’ or ‘three times’ daily. This result was similar before and after the intervention. The recommended frequency for toothbrushing is two to three times per day [2], which means most participants were following the recommendation.

The responses regarding brushing time were particularly noteworthy. Specifically, the proportion of participants selecting “three minutes” as their brushing time increased significantly after the intervention when compared to the baseline measurements. Conversely, the percentage of participants who chose answers like “less than two minutes” or “I don’t know” saw a notable decline. This shift suggests that participants extended their brushing time, with more of them consciously timing their brushing sessions to last for the recommended three minutes. This behavioral change is likely linked to the observed reduction in PHP scores, reflecting improved oral hygiene.

Previous research supports this trend, showing that as brushing time increases, plaque scores tend to decrease [34]. In this study, the considerable improvement in brushing time may be attributed to the use of Qscan or disclosing agents during the training sessions. These tools provided participants with immediate feedback, showing areas where plaque remained even after brushing. By encouraging participants to brush for a full three minutes and demonstrating that not all plaque was removed within that time, the program raised participants’ awareness about the need to invest adequate time in brushing their teeth. This direct, visual feedback could have significantly contributed to fostering better oral hygiene practices among the children.

Such interventions prompted the children to focus more on the thoroughness of their brushing rather than simply completing the task quickly. This outcome aligns with the broader understanding that extending brushing time can lead to more effective plaque removal and, consequently, better oral health outcomes. In light of these findings, it seems clear that tools like Qscan play a valuable role in educating children on the importance of brushing for the recommended time, reinforcing good habits, and ultimately leading to long-term improvements in oral health.

The degree of care about oral hygiene also shows that the number of “None” responses decreased compared to before participating in the study, and the number of “High” responses increased significantly, indicating that improvement in attitudes has been achieved through intervention. This aligns with the results of earlier studies that state that oral hygiene education programs are effective in improving oral health attitudes [25].

Among the responses to which of the two methods they prefer if they take toothbrushing training again, 43.6% said they prefer Qscan. Participants who said they preferred Qscan cited “Easily noticeable” as the most preferred reason. This result is because Qscan’s intraoral observation can easily show red fluorescence compared to color, while the disclosing agent can cause confusion for children by dyeing adjacent oral mucous membranes or tongues as well as dental plaques that need to be removed.

Recently, several studies have compared the quantitative evaluation of dental plaque using QLF images and disclosing agents. One study reported that, in patients with multibracket orthodontic appliances, QLF images showed lower plaque amounts than those detected by disclosing agents, suggesting that the quantification of plaque using QLF images may not be reliable [37]. Another study found that newly formed plaque exhibited less fluorescence than mature plaque, and the amount of plaque detected on the labial surfaces of molars was less than that measured by disclosing agents [38].

Although there are rather negative findings regarding QLF technology, a study also demonstrates a strong positive correlation between the intensity of red fluorescence in QLF images and stained plaque [39]. Even in studies that reported negative results, patients highly rated the convenience and user-friendliness of QLF images, indicating a preference for this method [37]. Similarly, our research highlights the efficacy of QLF technology in oral hygiene management and education, suggesting that QLF can be conveniently utilized in daily practice, which is significant.

There are several limitations to this study design. Since the wash-out period was set rather short, the pre-brushing PHP score measured on the third visit was lower than the first visit’s pre-brushing PHP score. The difference in PHP score before and after training may have been smaller than that of the first training by conducting a second training at a time when the wash-out was not sufficiently done. Secondly, the absence of a control group without oral hygiene education means we cannot assess the individual effects of the disclosing agent and Qscan. However, since this is a crossover design and each group has about the same assigned person, the effects of oral hygiene education using the disclosing agent and Qscan can be compared with each other, suggesting that the findings hold significance. Thirdly, we calculated the sample size to be 42 and initially recruited 58 participants, taking potential dropouts into account. Although we secured more than 42 participants by the mid-point of the study, we ultimately ended the clinical trial with 39 participants due to unexpected dropouts towards the end. However, in the context of a crossover clinical trial, it is important to note that all 39 participants used both the disclosing agent and Qscan rather than being split into two separate groups. Therefore, this does not significantly undermine the representativeness of the findings.

There were also limitations to using the Qscan device. Qscan is optimized for detecting red fluorescence in a dark environment, making it challenging to observe the red fluorescence clearly in a bright environment during the daytime. In addition, when children use Qscan to check their dental conditions, they have to put enough force on their lips or recline their lips, which is difficult to train. It would have been better if we added elements that can function as retractors to Qscan equipment. In addition, the small sample size limited the ability to generalize the results, and the short observation period made it difficult to assess the long-term effects of the educational program.

## 5. Conclusions

The QLF technology-based device, Qscan, demonstrated a reduction in plaque index when used for oral hygiene education for Korean children aged 6–11 years. However, no significant difference was observed when compared to the disclosing agent. The educational effects of Qscan were similar to those of the disclosing agent, and its ease of use makes Qscan a valuable supplementary tool for motivating children in oral hygiene education.

## Figures and Tables

**Table 1:** Variables of oral hygiene status in the two methods.

	Group	Pre-Brushing	Post-Brushing	Follow-Up	*p*-Value
PHP score	Qscan	1.58 ± 0.70	0.76 ± 0.39	1.08 ± 0.52	0.113
	Disclosing agent	1.71 ± 0.55	1.00 ± 0.43	1.20 ± 0.50	
SHS	Qscan	1.31 ± 1.78	0.49 ± 1.10	0.69 ± 1.03	0.379
	Disclosing agent	1.77 ± 1.72	0.64 ± 1.22	0.72 ± 1.02	
ΔR30 (%)	Qscan	2.05 ± 3.77	0.62 ± 1.63	0.82 ± 1.34	0.352
	Disclosing agent	2.74 ± 3.66	1.00 ± 2.53	1.08 ± 2.11	
ΔR120 (%)	Qscan	0.46 ± 1.39	0.03 ± 0.16	0.03 ± 0.16	1.000
	Disclosing agent	0.41 ± 0.88	0.05 ± 0.32	0.05 ± 0.32	

*p*-values from the between-group effect of repeated-measures ANOVA. Each values were the mean ± SD. PHP: patient hygiene performance; SHS: simple hygiene score.

**Table 2:** Time impact on oral hygiene status variables in the two methods.

	Source	F	*p*-Value
PHP score	Time	117.216	<0.001 *
	Time × Method	0.827	0.439
SHS	Time	20.968	<0.001 *
	Time × Method	0.949	0.374
ΔR30 (%)	Time	14.620	<0.001 *
	Time × Method	0.237	0.693
ΔR120 (%)	Time	9.500	<0.001 *
	Time × Method	0.089	0.774

* *p* < 0.05, *p*-values from repeated-measures ANOVA, Bonferroni post hoc test. PHP: patient hygiene performance; SHS: simple hygiene score.

**Table 3:** Responses from the questionnaire survey at baseline and post-intervention for participants.

Question	Answer	Baseline		Post-Intervention		
		n	%	n	%	*p*-Value
Number of times to brush teeth per day (n)	1	2	5.1	1	2.6	0.741
	2	12	30.8	12	30.8	
	3	21	53.8	22	56.4	
	4	4	10.3	4	10.3	
Brushing time (min)	Less than 2	7	17.9	4	10.3	0.02 *
	2	11	28.2	10	25.6	
	3	9	23.1	16	41	
	More than 4	1	2.6	2	5.1	
	I don’t know	11	28.2	7	17.9	
Degree of concern about oral hygiene	None	4	10.3	2	5.1	0.011 *
	Low	21	53.8	16	41	
	High	14	35.9	21	53.8	

* *p* < 0.05, *p*-values from Fisher’s exact test.

**Table 4:** Preferred method for toothbrushing instruction.

Preferred Method	N	%
Qscan	17	43.6
Disclosing agent	3	7.7
Both of them	14	35.9
None of them	1	2.6
I don’t know	4	10.3

**Table 5:** Reasons why such a method is preferred (multiple choice).

Reason for Preference	Qscan	Disclosing Agent
Clean	3	0
Easily noticeable	13	2
Convenient	9	1

## Data Availability

The data presented in this study are available upon request from the corresponding author.
